# First cytogenetic analysis of *Ichthyoelephas humeralis* (Günther, 1860) by conventional and molecular methods with comments on the karyotypic evolution in Prochilodontidae

**DOI:** 10.3897/CompCytogen.v10i4.9858

**Published:** 2016-11-18

**Authors:** Mauro Nirchio Tursellino, Duílio Mazzoni Zerbinato de Andrade Silva, César Quezada Abad, Wilmer Arnoldo Moreira Blacio, Omar Rogerio Sánchez Romero, Claudio Oliveira

**Affiliations:** 1 Universidad Técnica de Machala, Facultad de Ciencias Agropecuarias, Av. Panamericana Km 51/2, Vía Machala Pasaje, El Oro, Ecuador; 2 Universidad de Oriente, Escuela de Ciencias Aplicadas del Mar, Boca de Río, Península de Macanao, Estado Nueva Esparta, Venezuela; 3 Laboratório de Biologia e Genética de Peixes, Instituto de Biociências de Botucatu, Universidade Estadual Paulista (UNESP), Departamento de Morfologia, Distrito de Rubião Junior, Botucatu, São Paulo, Brazil. CEP: 18618-689

**Keywords:** Karyotype, evolution, Prochilodontidae, Fluorescent *in situ* hybridization, NORs

## Abstract

We used conventional cytogenetic techniques (Giemsa, C-banding, Ag-NOR), and fluorescent *in situ* hybridization (FISH) with 5S and 18S rDNA probes to investigate the karyotype and cytogenetic characteristics of *Ichthyoelephas humeralis* (Günther, 1860) from Ecuador. The specimens studied have a karyotype with 2n=54 biarmed chromosomes (32 M + 22 SM) and C-positive heterochromatin located on the centromeric, pericentromeric, interstitial, and terminal regions of some chromosomes. The nucleolus organizer regions occurred terminally on the long arm of chromosome pair 2. FISH confirmed the presence of only one 18S rDNA cluster with nonsyntenic localization with the 5S rDNA. Cytogenetic data allow us to refute the earlier morphological hypothesis of a sister relationship between *Semaprochilodus* Fowler, 1941 and *Ichthyoelephas* Posada Arango, 1909 and support the molecular proposal that *Ichthyoelephas* is a sister group to the monophyletic clade containing *Prochilodus* Agassiz, 1829 and *Semaprochilodus*.

## Introduction

The fish family Prochilodontidae includes 21 valid species, with three recognized genera: *Ichthyoelephas* Posada Arango, 1909, *Prochilodus* Agassiz, 1829 and *Semaprochilodus* Fowler, 1941 (Castro and Vari 2004, Eschmeyer and Fong 2016). These species constitute a valuable resource of commercial and subsistence freshwater fish distributed throughout the South American countries, except Chile (Lowe-McConnell 1975, Goulding 1981, Flecker 1996). *Ichthyoelephas* live in the Andean rivers west of Colombia and Ecuador. *Prochilodus* is present in all major South American river systems on both sides of the Andes, and *Semaprochilodus* is broadly distributed east of the Andes along the Amazon, Tocantins and Orinoco basins and some coastal rivers draining the Guiana Shield (Castro and Vari 2004).

Cytogenetic studies conducted thus far in Prochilodontidae are limited to *Prochilodus* (8/13 species karyotyped) and *Semaprochilodus* (4/6 species karyotyped). Those works revealed a conserved karyotype composed of 54 metacentric-submetacentric chromosomes with a fundamental number (FN)=108 (Arai 2011), with a heteromorphic ZW pair reported only in *Semaprochilodus taeniurus* (Valenciennes, 1817) karyotype (Terencio et al. 2012a). However, no cytogenetic data are available for the two *Ichthyoelephas* species, *Ichthyoelephas longirostris* (Steindachner, 1879), and *Ichthyoelephas humeralis* (Günther, 1860)

In this research, for the first time we used the available karyotyping techniques, including Giemsa-staining, Ag-staining, C-banding, and localization of 18S rDNA and 5S rDNA to investigate the cytogenetic characteristics of *Ichthyoelephas humeralis*.

## Methods

We analyzed nineteen specimens of *Ichthyoelephas humeralis* (undetermined sex) collected with seine nets in the channels fed by the Babahoyo River (2°00'41.4"S 79°47'00.1"W), which supply water to the rice plantations of Samborondon, Guayas Province, Ecuador. Voucher specimens were fixed in 10% formalin and deposited in the fish collection of the Laboratório de Biologia e Genética de Peixes, UNESP, Botucatu (São Paulo State, Brazil) (collection numbers LBP 19326), and Universidad Técnica de Machala (collection numbers UTMach-00184).

We obtained kidney cell suspensions from fish injected intramuscularly with yeast-glucose solution for mitosis stimulation 24 hours before injecting colchicine (Lee and Elder 1980). Chromosome preparations were obtained injecting 0.0125% colchicine intraperitoneally (1.0 ml/100 g body weight) 50 min before sacrificing, as described by Nirchio and Oliveira (2006). Before being sacrificed, the specimens received a numbing overdose of Benzocaine (250 mg/L) until the cessation of opercular movements (AVMA 2013). Mitotic chromosome preparations were obtained by the conventional air-drying method, as described in Nirchio and Oliveira (2006).

We analyzed a minimum of 10 metaphases per sample using all investigative techniques separately. Silver (Ag) staining revealed active nucleolus organizer regions (NORs), as described by Howell and Black (1980) sequentially after Giemsa staining (Rábová et al. 2015). We obtained C-bands following the method of Sumner (1972).

Physical mapping of major and minor ribosomal genes on the chromosomes was performed by fluorescence *in situ* hybridization (FISH) following the method described by Pinkel et al. (1986). Both major (18S rDNA) and minor (5S rDNA) ribosomal probes were isolated from DNA extracted from samples of the same species by PCR. The probe for rDNA was obtained using the primers 18S6F (5’CTCTTTCGAGGCCCTGTAAT3’) and 18S6R (5’CAGCTTTGCAACCATACTCC3’) (Utsunomia et al. 2016). We accomplished the labeling of this probe with Digoxigenin-11-dUTP (Roche Applied Science), and hybridization signal detection was performed using Anti-Digoxigenin-Rhodamine (Roche Applied Science). To obtain the 5S rDNA probe, we used the primers 5SF (5’TCAACCAACCACAAAGACATTGGCAC3’) and 5SR (5’TAGACTTCTGGGTGGCCAAAGGAATCA3’) (Pendás et al. 1994). This probe was labeled with Biotin-16-dUTP (Roche Applied Science), and hybridization signal detection was performed using conjugated Avidin-Fluorescein (FITC).

We photographed the mitotic chromosomes using a Motic B410 microscope equipped with a Motic Moticam 5000C digital camera. The chromosomes were classified as metacentric (M) or submetacentric (SM) according to the arm ratio criteria (Levan et al. 1964). FISH metaphases were photographed with an Olympus BX61 photomicroscope equipped with a DP70 digital camera. Images were digitally processed with ADOBE PHOTOSHOP CC 2015.

## Results

**Figure 1.**
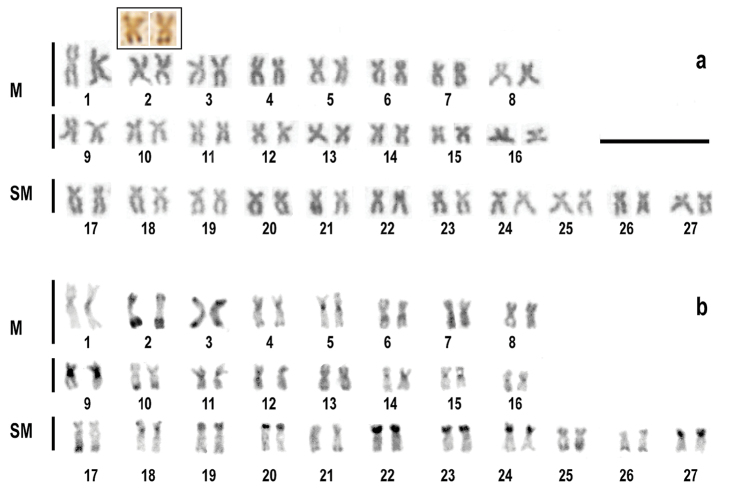
Karyotypes of *Ichthyoelephas humeralis* after Giemsa staining (**a**) and C-banding (**b**). Ag-NORs inbox. Bar = 10 µm

The karyotype of *Ichthyoelephas humeralis*, obtained from 247 metaphases achieved from the 19 analyzed individuals, revealed a modal diploid number of 2n=54 composed of 32 M and 22 SM (Fig. 1a). Chromosomes of metacentric and submetacentric series decrease uniformly in size, making it difficult to identify homologous chromosomes. Only the metacentric chromosome pair 1, the largest in the complement, can be identified unequivocally in the metacentric series. Pair 1 consistently showed a variation in size in all the recorded metaphases of all fishes studied (Fig. 1a). Chromosomal differences between sexes were not observed.

C-banding showed heterochromatic blocks located in the centromeric region of pairs number 4, 5, 9, 11, 14, 15, 16, 18. C-bands appeared in the terminal regions of pairs 2, 3, 10, 17, 19, 20, 22, 23, 24, 25, 26, 27; and, in the pericentromeric regions of pairs 1 and 9; and interstitially on pair 6. Chromosomes 7, 8, 12, 13, and 21 did not show typical constitutive heterochromatin marks (Fig. 1b). Discrete C-banding marks in the terminal regions of the long arm of chromosome pair N° 2 were coincident with the Ag-NORs (Fig. 1a).

**Figure 2.**
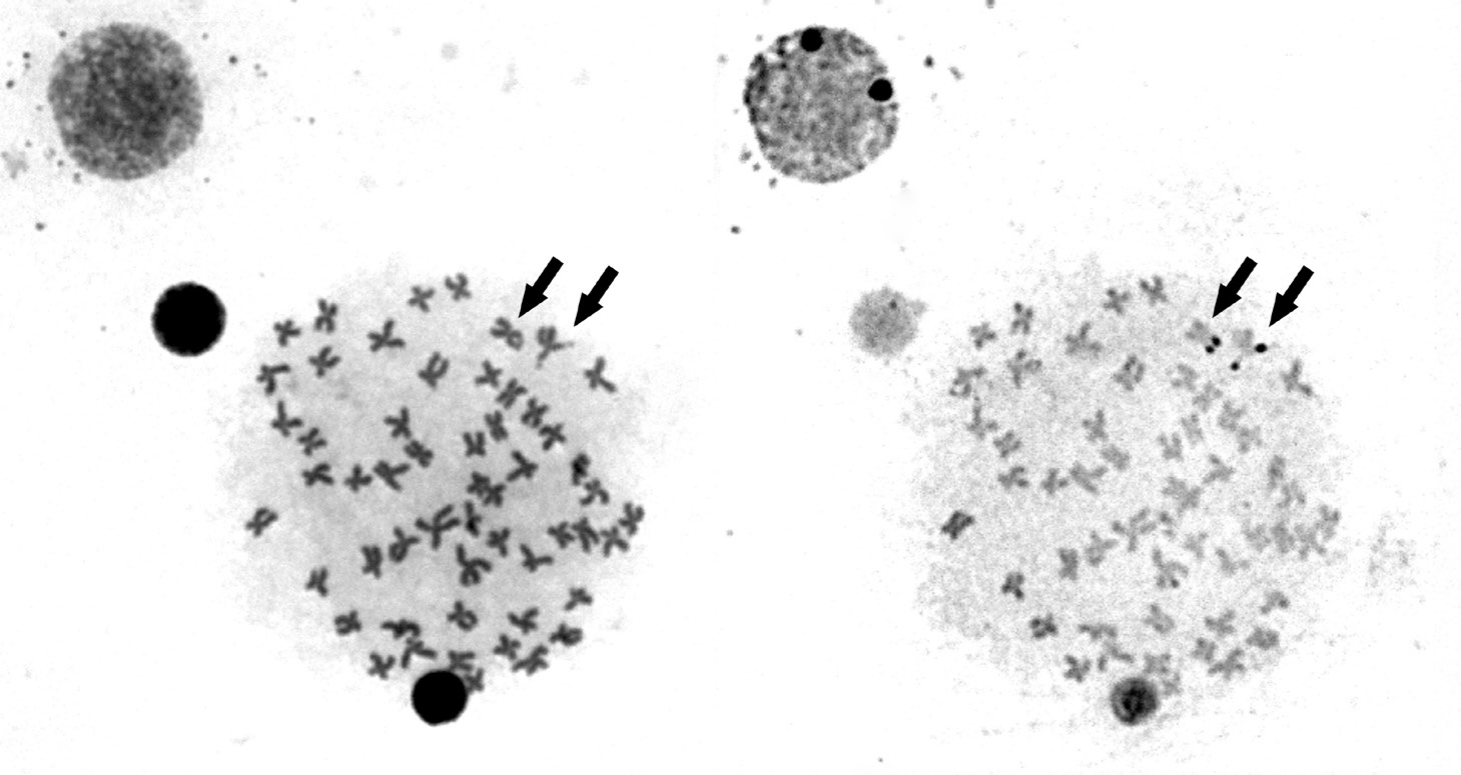
Ag-NOR staining on metaphase chromosomes of *Ichthyoelephas humeralis* after Giemsa staining (arrows show the NOR-bearing chromosomes).

**Figure 3.**
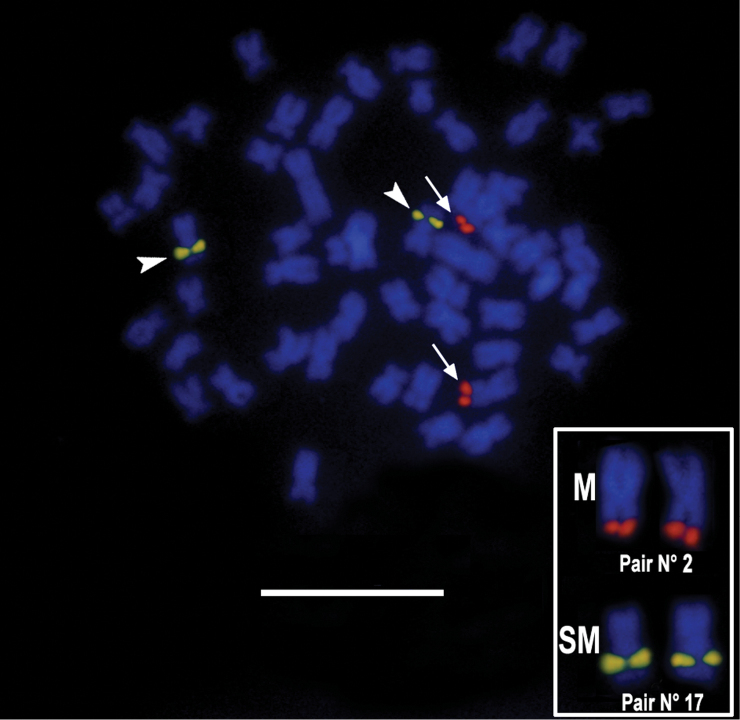
Double FISH staining of metaphase chromosomes of *Ichthyoelephas humeralis* (arrows show the 18S rDNA, and arrowheads show the 5S rDNA); inbox details of chromosome bearing 5S and 18S rDNA. Bar = 10 µm.

Impregnation with AgNO_3_ after Giemsa staining revealed only one pair of active nucleolus organizer regions (Ag-NOR), located on the tips of the long arms of a metacentric chromosome possessing an evident secondary constriction (Fig. 2). This chromosome was identified as pair 2 in the karyotype (Fig. 1a). FISH with 18S rDNA probe produced bright Avidin-Fluorescein (FITC) signals only on the tips of the long arms of chromosome pair 2, which indicates that the species does not possess additional NOR-sites (Fig. 3). FISH with 5S rDNA probe produced interstitial FITC signals on the long arm of a chromosome pair, apparently the largest of the SM series (Pair N° 17), thus demonstrating by double FISH that both ribosomal gene clusters are located on different chromosomes (Fig. 3).

## Discussion

By adding the chromosome information on *Ichthyoelephas humeralis* reported herein to the Prochilodontidae database, the number of the species of the family so far cytogenetically analyzed rises to 13, out of the 21 currently recognized valid species (Eschmeyer and Fong 2016). Cytogenetic studies conducted with 12 representatives of the genera *Prochilodus* and *Semaprochilodus* show that they have an evolutionarily conserved karyotype with 2n=54 biarmed elements, composed of 40 metacentric and 14 submetacentric chromosomes with a fundamental number (FN)=108 (Arai 2011). The exception lies in a few *Prochilodus* species or populations showing intra and interpopulation karyotype variation related to supernumerary B chromosomes (Pauls and Bertollo 1983, 1990, Oliveira et al. 2003, Gras et al. 2007, Penitente et al. 2015). The present data about *Ichthyoelephas humeralis* confirm the occurrence of a conservative chromosome diploid complement and fundamental number in Prochilodontidae. Notwithstanding, its karyotypic formula differs in the number of metacentric and submetacentric chromosomes suggesting that pericentromeric inversions occurred in four submetacentric pairs changing the number of metacentric chromosomes from 32 to 40 or vice-versa. These events occurred after the divergence of *Ichthyoelephas* from *Prochilodus* and *Semaprochilodus* (Melo et al., 2016) since these two groups belong to different lineages described in Prochilodontidae.

C-banding in *Ichthyoelephas humeralis* revealed constitutive heterochromatin in the centromeric, pericentromeric, interstitial, and terminal regions. These characteristics are difficult to compare quantitatively to other Prochilodontidae species. Nevertheless, this heterochromatin distribution is different regarding the particular pattern in other species of Prochilodontidae, which show heterochromatin typically restricted to the centromeric and pericentromeric regions of their chromosomes (Oliveira et al. 2003, Vicari et al. 2006, Terencio et al. 2012b, Voltolin et al. 2013).

Ribosomal sites in Prochilodontidae (5S and 18S ribosomal clusters) are syntenic, commonly located in the interstitial position on chromosome pair 2 in all species of *Prochilodus* and *Semaprochilodus* analyzed (Pauls and Bertollo 1990, Oliveira et al. 1997, 2003; Venere et al. 1999, Cavallaro et al. 2000, Maistro et al. 2000, Jesus and Moreira-Filho 2003, Hatanaka and Galetti Jr. 2004, Artoni et al. 2006, Vicari et al. 2006, Gras et al. 2007, Voltolin et al. 2009, 2013, Jorge et al. 2011, Terencio et al. 2012a, 2012b, Penitente et al. 2015).

The localization of ribosomal clusters on distinct chromosome pairs in *Ichthyoelephas humeralis* with the18S rDNA terminally located on pair 2 and the 5S rDNA interstitially positioned on pair 17, suggests the occurrence of at least two chromosome reorganization events when *Ichthyoelephas*, *Prochilodus* and *Semaprochilodus* diverged from their common ancestor: 1) a paracentromeric inversion to explain the displacement of the 18S rDNA cluster from a terminal to an interstitial position or vice-versa, and 2) a translocation of the ribosomal 5S rDNA site from its bearing chromosome to an 18S rDNA bearing chromosome or vice-versa.

The most comprehensive molecular phylogenetic study in Prochilodontidae based on mitochondrial and nuclear loci (Melo et al. 2016) provides evidence supporting the position of *Ichthyoelephas* as a sister group to the clade of *Prochilodus* and *Semaprochilodus*. Curimatidae and Chilodontidae are sister groups to Prochilodontidae (Oliveira et al. 2011, Melo et al. 2016). Data on NORs in Chilodontidae and Curimatidae show that species in these families have only one NOR-bearing chromosome pair, usually a large metacentric with NORs in the terminal position (Martins et al. 2000, De Rosa et al. 2006, Rodrigo et al. 2008, Venere et al. 2008, Arai 2011) as observed in *Ichthyoelephas humeralis*, subject of this study. FISH experiments with species of Curimatidae show that the 18S rDNA sites are coincident with the Ag-NORs, and 5S rDNA are found on different chromosomes in interstitial positions in all species analyzed (De Rosa 2006, 2007).

Castro and Vari (2004) proposed a close relationship between *Semaprochilodus* and *Ichthyoelephas* based on morphological studies. This result was refuted by Melo et al. (2016), who, based on molecular data, observed a close relationship between *Semaprochilodus* and *Prochilodus*. As described above, the present cytogenetic data show that *Prochilodus* and *Semaprochilodus* share several chromosomal characteristics, such as the syntenic location of 5S and 18S ribosomal genes, constitutive heterochromatin distribution pattern, and karyotypic formula. All these chromosomal characteristics are not observed either in *Ichthyoelephas*, Curimatidae, or Chilodontidae. Thus, cytogenetic data corroborated the hypothesis of Melo et al. (2016), whereby *Prochilodus* and *Semaprochilodus* are closely related and may be sister groups to *Ichthyoelephas humeralis*. Further studies should be performed to establish whether *Ichthyoelephas humeralis* shares the chromosome characteristics with the only additional species in the genus: *Ichthyoelephas longirostris*.

The results described here demonstrate the usefulness of conventional and molecular cytogenetic techniques as tools for understanding the evolutionary history in Prochilodontidae suggesting the occurrence of some micro and chromosomal macrostructural reorganization events in the ancestral karyotype wherefrom *Ichthyoelephas* arose as a clade that diverged from the ancestor of their sister group *Prochilodus*-*Semaprochilodus* approximately 12 million years ago (Melo et al. 2016).
